# A Comparison of the Effectiveness of Sodium Stibogluconate Monotherapy to Sodium Stibogluconate and Paromomycin Combination for the Treatment of Severe Post Kala Azar Dermal Leishmaniasis in South Sudan – A Retrospective Cohort Study

**DOI:** 10.1371/journal.pone.0163047

**Published:** 2016-09-22

**Authors:** Charles Abongomera, Francis Gatluak, Jozefine Buyze, Koert Ritmeijer

**Affiliations:** 1 Médecins Sans Frontières, Amsterdam, The Netherlands; 2 Médecins Sans Frontières, Lankien, South Sudan; 3 Institute of Tropical Medicine, Antwerp, Belgium; Taibah University, SAUDI ARABIA

## Abstract

**Background:**

Post-kala-azar dermal leishmaniasis (PKDL) is a common dermatological complication following successful treatment of Visceral Leishmaniasis (VL) caused by *Leishmania donovani*. PKDL presents as macular, papular, nodular or mixed skin rash on sun-exposed body parts. Patients are not ill unless there are complications due to mucosal involvement or ulceration. As PKDL in East Africa is typically self-healing, and treatment is long and with significant adverse events, only severe and complicated cases are treated. Studies to determine optimal treatment of PKDL are rare and based on small cohorts. Since 1989, Médecins Sans Frontières is treating severe PKDL within VL treatment programmes in South Sudan. Treatment was initially with sodium stibogluconate (SSG) monotherapy and since 2002 with a combination of SSG and paromomycin (PM). SSG monotherapy (20 mg/kg/day for a minimum of 30 days) was provided in primary health units, and the combination of PM (15 mg sulphate/kg/day for 17 days) plus SSG (30 mg/kg/day for a minimum of 17 days) was provided in secondary health facilities.

**Methodology/Principal Findings:**

By retrospective analysis of routinely collected programme data we compared the effectiveness (outcome and treatment duration) of both regimens. Between 2002 and 2008, 422 patients with severe PKDL were treated; 343 received SSG and 79 SSG/PM combination. The cure rate was significantly better with combination treatment when compared to monotherapy (97% *vs*. 90%; odds ratio [OR], 7.6; p = 0.02), treatment duration was shorter (mean 34 days *vs*. 42 days; p = 0.005), and defaulter rate was lower (3% *vs*. 9%; OR, 0.3; p = 0.03). There was no significant difference in death rate (0% *vs*. 1%; p = 0.5).

**Conclusions/Significance:**

We found that SSG/PM combination therapy resulted in more favourable outcomes than SSG monotherapy. An additional advantage is the lower cost of the combination therapy, due to the shorter treatment duration. A combination of SSG and PM is therefore a suitable option for the treatment of PKDL in East Africa.

## Introduction

East Africa is one of the most endemic regions in the world for visceral leishmaniasis (VL) or kala-azar with an estimated annual incidence of 30,000 to 60,000 cases [1]. Post-kala- azar dermal leishmaniasis (PKDL) is a common dermatological complication of VL, which typically occurs after successful treatment of VL caused by *Leishmania donovani*. However, in rare cases PKDL may also present with simultaneous VL infection (para-KDL) or even without history of VL [2–4].

The incidence of PKDL is not exactly known because systematic follow up of VL patients after treatment is rare. The incidence seems to differ greatly between countries: 56% to 62% in Eastern Sudan, 14% in Ethiopia and 0.05% to 30% in Kenya [3,5–7]. The PKDL incidence in South Sudan is not known, but is considered significant. The probable risk factors for developing PKDL are malnutrition, HIV infection, young age, drug used for VL treatment and inadequate dosage of VL treatment regimens [3,4,6,8].

PKDL patients present with a skin rash which may be macular, papular, nodular or mixed. The rash usually starts around the mouth and may spread to the face, trunk, arms, and the rest of the body [2,4,5,9]. In East Africa, mixed maculo-papular rash is the most common. Lesions are usually symmetrical and not itchy, and there is no loss of sensation [2,10], which helps in differentiation from other common skin infections such as leprosy. PKDL patients with grade 1 and 2 non-severe are not as ill compared to those with grade 3 and 2-severe who experience significant discomfort due to mucosal involvement, ulceration or disfiguring nodules. The PKDL rash may last for weeks or months, and in a minority of patients become generalized and severe with mucosal lesions in the mouth, nose or eyes, causing potentially severe complications [4].

Ismail *et*. *al*. demonstrated that *Leishmania* parasites could be detected in up to 88% of biopsies from PKDL skin lesions [11]. Furthermore, there is also evidence that the sandfly vector may become infected with *Leishmania* while taking a blood meal and it is assumed that PKDL patients may play an important role in transmission of VL (anthroponotic transmission)[12,13]. Historical data suggest that VL occurs in cycles with epidemic waves, followed by periods of seemingly low transmission. It has been suggested that chronic PKDL patients may play an important role as a reservoir in subsequent upsurges of VL cases [13].

In East Africa, PKDL is not routinely treated since the majority of cases (85%) heal spontaneously within 1 year, and therefore treatment is restricted to severe and complicated PKDL, and lesions that have remained for more than 6 months [4,9,14]. Clinical studies to determine the optimal treatment for PKDL are rare and based on small cohorts. Current treatment is on compassionate ground and not standardised. In East Africa, sodium stibogluconate (SSG) is commonly used but is potentially toxic, with daily painful injections, and treatment is long (30–60 days) leading to prolonged hospital stay [2–4,9,15]. Liposomal amphotericin B has been shown to be safe and effective but the long treatment course (20 days) makes this regimen prohibitively expensive [16].

Since 1989, Médecins Sans Frontières (MSF) is treating severe PKDL within VL treatment programmes in South Sudan. Treatment of PKDL was initially with SSG monotherapy, and since 2002 a combination therapy of SSG and paromomycin (PM) was used in secondary level care facilities. By retrospective analysis of routinely collected programme data we compared the effectiveness (outcome and treatment duration) of SSG monotherapy and SSG/PM combination therapy.

## Methods

We conducted a retrospective cohort study using routinely collected programme data. The study sites were located in the South Sudan VL endemic states of Jonglei, Upper Nile and Unity. MSF runs primary health care units (PHCUs), primary health care centres (PHCCs) and hospitals where PKDL, VL and other illnesses are treated. Medical doctors worked in the PHCCs and hospitals while community health workers (CHWs) and nurses run the PHCUs. The diagnosis and treatment decision in the PHCCs and hospitals were made by a medical doctor, and in the PHCUs by a health worker, who had been trained by an MSF doctor. Complicated medical cases were referred by the PHCUs to the PHCCs and hospitals. The PHCU staff were also able to consult the doctors by radio for advice on complicated medical cases.

We included all patients diagnosed with severe or complicated PKDL (grade 3 and disfiguring grade 2) between 2002 and 2008, that had been treated with either SSG or SSG/PM combination and their treatment outcomes and duration reported. We excluded patients that had been treated with both SSG and SSG/PM combination.

A severe or complicated PKDL case was diagnosed clinically because of its specific signs and symptoms, further supported by a history of VL or concurrent VL and based on the grading system shown in Table 1 [3].

**Table 1:** Post-kala- azar dermal leishmaniasis grading system.

	Distribution of the rash
**Grade 1**	Scattered maculopapular or nodular rash on the face, with or without lesions on the upper chest or arms
**Grade 2**	Dense maculopapular or nodular rash covering most of the face and extending to the chest, back, upper arms and legs, with only scattered lesions on the forearms and legs
**Grade 3**	Dense maculopapular or nodular rash covering most of the body, including the hands and feet; the mucosa of the lips and palate may be involved and crusting and scaling occurs

Severe or complicated PKDL cases are those that experience significant discomfort due to mucosal involvement, ulceration or disfiguring nodules. Only severe or complicated PKDL was treated. Severe grade 2 was distinguished from other grade 2 by the disfiguring element, usually presenting as black nodules on and around the nose. PKDL cure was defined as flattening of lesions, improvement of dyschromia and healing of complications [14].

Patients were treated either as per World Health Organisation (WHO) protocol with SSG (Albert David Ltd., Kolkata, India) at a dose of 20 mg/kg/day (minimum daily dose 200 mg, no maximum dose) administered by intramuscular injection or as per MSF compassionate protocol with SSG and PM (Gland Pharma Ltd., Hyderabad, India). In the MSF compassionate protocol, SSG was administered as above while the PM dosage was 15 mg sulphate/kg/day (11 mg/base/kg/day) administered intramuscularly for 17 days (minimum daily dose 50 mg, maximum dose 1,150 mg) [14]. In this MSF compassionate protocol, if there was no significant improvement at 17 days and no contraindications to SSG, SSG alone was continued beyond 17 days until there was significant improvement. During treatment, daily monitoring of the evolution of the PKDL lesions was done.

As in the study period (2002–2008) the safety of SSG/PM combination therapy for VL was still under study by the Drugs for Neglected Diseases Initiative (DNDi), it was decided that SSG/PM would only be provided under supervision of a medical doctor. Therefore combination therapy was only started in the hospitals and PHCCs under supervision of a doctor and monotherapy was started in the PHCUs under supervision of the CHWs or nurses.

Demographic details, date of diagnosis, duration of illness, nutritional status, PKDL grade, PKDL treatment regimen and discharge date of patients were documented. Data from the patient files were entered into a computerised excel data collection sheet. Inaccurate records were systematically detected and corrected or removed by the MSF staff and researchers. Statistics generated by the monthly reports were compared with data records at the treatment facilities. Missing and conflicting information was rectified. A Microsoft excel data set containing information from all health facilities was then created.

This study is based on the compiled excel data set containing data from 2002 to 2008 from the different health facilities. Data were analysed using STATA 13 software [StataCorp. 2013. *Stata Statistical Software*: *Release 13*. College Station, TX: StataCorp LP]. Odds ratios (OR) for the different outcomes (cured, defaulted, died) were calculated for the different variables (PKDL treatment regimen, sex, age, duration of illness and nutritional status). Differences in proportions for the different outcomes were compared using the Fisher’s exact test while differences in means of treatment duration were compared using the unpaired (two-sample) t-test. To control for confounding exact logistic and multiple linear regression were performed. Exact logistic regression was performed because there were few patients in some subgroups. The level of significance was set at p<0.05 and the 95% confidence intervals of the differences were calculated.

This was an anonymous retrospective data analysis of routine programme data, patient written informed consent was not necessary and it was exempted from full formal ethical approval as per MSF International Ethics Review Committee criteria.

## Results

**Fig 1:**
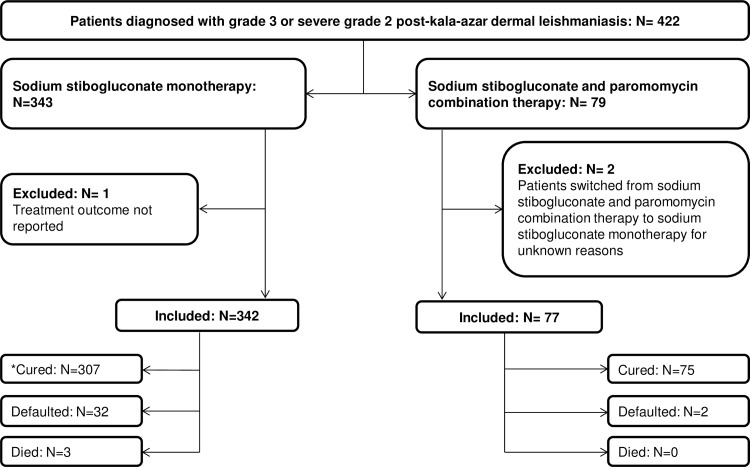
Flow diagram showing the number of patients in the study and their outcomes. * Treatment duration was not reported in one patient. This patient was excluded from the analysis on treatment duration.

Between 2002 and 2008, 422 patients were diagnosed with grade 3 or severe grade 2 PKDL and treated at the different health facilities. Three hundred and forty three patients (81.3%) were treated with SSG and 79 (18.7%) with SSG/PM combination. Two patients were switched from the combination treatment to SSG monotherapy after 4 and 5 days respectively. The reasons for the switch were unknown and they were excluded from the study. Treatment outcome and treatment duration were not reported for 2 different patients on SSG monotherapy. These 2 patients were excluded from the analysis on treatment outcome and duration respectively. Three hundred and forty two patients (99.7%) treated with SSG and 77 patients (97.4%) treated with SSG/PM were included in the study (Fig 1).

A total of 6,888 VL patients were treated in the study period but the incidence of PKDL was not known. This is because the majority of PKDL cases (notably grade 1 and 2-non severe) do not require treatment and therefore did not go to the health facilities thereby remaining unidentified.

In the study group, there were 210 males (50.1%), 320 PKDL grade 3 cases (76.4%) and 200 malnourished cases (47.7%). The median age at admission was 5 years (IQR 2–16.5) and the mean duration of illness at admission was 2.3 (± 2.2) months. Three hundred and eighty two patients were cured (91.2%), 34 defaulted (8.1%) and 3 died (0.7%) as shown in Table 2.

**Table 2:** Demographic and baseline clinical characteristics of patients with post-kala-azar dermal leishmaniasis treated by Médecins Sans Frontières in South Sudan from 2002 to 2008.

	Cured	Defaulted	Died	Total
N	382	34	3	419
**Sex, n (%)**				
Male	193 (50.5)	15 (44.1)	2 (66.7)	210 (50.1)
Female	183 (47.9)	18 (52.9)	1 (33.3)	202 (48.2)
Missing	6 (1.6)	1 (3.0)	0 (0.0)	7 (1.7)
**Age (years),** median (IQR)	5 (2–16)	12 (1.5–22)	1 (0.7–7)	5 (2–16.5)
**Age groups, n (%)**				
≤ 5 years	200 (52.4)	16 (47.1)	2 (66.7)	218 (52.0)
> 5 years	179 (46.8)	18 (52.9)	1 (33.3)	198 (47.3)
Missing	3 (0.8)	0 (0.0)	0 (0.0)	3 (0.7)
**Duration of illness (months),** mean (SD)	2.4 (2.3)	1.8 (1.4)	1.7 (2.0)	2.3 (2.2)
**Duration of illness, n (%)**				
≤ 2 months	134 (35.1)	16 (47.1)	2 (66.7)	152 (36.3)
> 2 months	214 (56.0)	16 (47.1)	1 (33.3)	231 (55.1)
Missing	34 (8.9)	2 (5.8)	0 (0.0)	36 (8.6)
**PKDL grade, n (%)**				
Grade 2	49 (12.8)	0 (0.0)	0 (0.0)	49 (11.7)
Grade 3	285 (74.6)	32 (94.1)	3 (100.0)	320 (76.4)
Missing	48 (12.6)	2 (5.9)	0 (0.0)	50 (11.9)
**Nutritional status, n (%)**				
Normal	163 (42.7)	14 (41.2)	1(66.7)	178 (42.5)
Mild malnutrition	21 (5.5)	1 (2.9)	0 (0.0)	22 (5.2)
Moderate malnutrition	83 (21.7)	8 (23.5)	0 (0.0)	91 (21.7)
Severe malnutrition	79 (20.7)	6 (17.7)	2 (33.3)	87 (20.8)
Missing	36 (9.4)	5(14.7)	0 (0.0)	41 (9.8)
**PKDL treatment regimen, n (%)**				
SSG	307 (80.4)	32 (94.1)	3 (100.0)	342 (81.6)
SSG/PM	75 (19.6)	2 (5.9)	0 (0.0)	77 (18.4)
**Treatment duration (days)**				
Mean (SD)	39.6 (14)	26.4 (13.4)	16.7 (11.7)	38.4 (14.5)
Missing, n (%)	1 (0.3)	0 (0.0)	0 (0.0)	1 (0.2)

IQR—Inter Quartile Range; SD—Standard deviation; PKDL—Post-kala-azar dermal leishmaniasis; SSG—Sodium stibogluconate; PM–Paromomycin

Eleven (22.4%) of the 49 patients with PKDL grade 2 and 54 (16.9%) of the 320 patients with PKDL grade 3 were treated with SSG/PM combination. The distribution of PKDL grades in the two treatment groups was not different (p = 0.22).

Seventy five of the 77 patients treated with SSG/PM and 307 of the 342 patients treated with SSG were cured as shown in Table 2. The cure rate was significantly higher in patients treated with SSG/PM (97.4% *vs*. 89.8%; adjusted OR, 7.58; 95% CI, 1.21–315.06; p = 0.02) as shown in Table 3.

**Table 3:** Odds ratios for cure, defaulting and death for patients with post-kala-azar dermal leishmaniasis treated by Médecins Sans Frontières in South Sudan from 2002 to 2008.

	Cure				Defaulting		Death	
	Crude OR (95% CI)	P	Adjusted OR (95% CI)	p	Crude OR (95% CI)	p	Crude OR (95% CI)	p
**Sex**								
Female	1.0	0.38	-	-	1.0	0.32	1.0	0.51
Male	1.18 (0.59–2.34)		-		0.79 (0.39–1.60)		1.93 (0.18–20.65)	
**Age (years)**								
> 5 years	1.0	0.38	-	-	1.0	0.32		0.54
≤ 5 years	1.18 (0.60–2.32)		-		0.79 (0.39–1.59)		1.82 (0.17–19.63)	
**Duration of illness (months)**								
≤ 2 months	1.0	0.09	1.0	0.36	1.0	0.14	1.0	0.35
> 2 months	1.69 (0.85–3.38)		1.48 (0.69–3.19)		0.63 (0.31–1.30)		0.33 (0.03–3.23)	
**Nutritional status**								
Moderate/severe malnutrition	1.0	0.44	-	-	1.0	0.52	1.0	0.46
Mild malnutrition/ normal status	1.14 (0.55–2.34)		-		0.95 (0.44–2.03)		0.44 (0.04–4.63)	
**PKDL treatment regimen**								
SSG	1.0	0.02	1.0	0.02	1.0	0.03	-	0.54
SSG/PM	4.28 (1.12–16.26)		7.58 (1.21–315.06)		0.26 (0.07–0.99)		-	

OR—Odds ratio; CI—Confidence interval; PKDL—Post-kala-azar dermal leishmaniasis; SSG—Sodium stibogluconate; PM–Paromomycin

The duration of illness at admission was not associated with cure (adjusted OR, 1.48; 95% CI, 0.69–3.19; p = 0.36). All patients with PKDL grade 2 were cured in both treatment groups. Among PKDL grade 3 patients, 52 (96.3%) were cured on SSG/PM treatment compared to 233 (87.6%) on SSG treatment (crude OR, 3.68; 95% CI, 0.94–14.49; p = 0.042).

Two of the 77 patients treated with SSG/PM and 32 of the 342 patients treated with SSG defaulted as shown in Table 2. The defaulter rate was significantly lower in patients treated with SSG/PM (2.6% *vs*. 9.4%; crude OR, 0.26; 95% CI, 0.07–0.99; p = 0.03) as shown in Table 3. In the group treated with SSG, 15.6% (5/32) of defaulters absconded during the first two weeks of treatment, 37.5% (12/32) during the second two weeks, and 46.9% (15/32) after the fourth week.

None of the patients treated with SSG/PM and 3 of the 342 patients treated with SSG died as shown in Table 2. There was no significant difference in death rate in the 2 treatment groups (0% *vs*. 0.9%, p = 0.5) as shown in Table 3.

Treatment duration was documented for 381 cured patients (99.7%). The mean treatment duration for patients on SSG/PM therapy was 33.7 (± 15.5) days compared to 41.1 (± 13.3) days for patients on SSG therapy. After adjusting for the duration of illness at admission, the treatment duration was shorter for patients treated with SSG/PM (-5.13 days; CI -8.73–-1.53; p = 0.005) as shown in Table 4.

**Table 4:** Treatment duration for patients with post-kala-azar dermal leishmaniasis treated by Médecins Sans Frontières in South Sudan from 2002 to 2008.

	Treatment duration (days)		Crude difference in means (95% CI)	p	Adjusted difference in means (95% CI)	P
	N = 381	Means (SD)				
**Sex, n (%)**						
Female	183 (48.0)	39.6 (13.9)	-0.2 (-3.1–2.6)	0.86		
Male	192 (50.4)	39.8 (14.2)				
Missing	6 (1.6)					
**Age (years), n (%)**						
≤ 5 years	199 (52.2)	40.1 (15.3)	0.9 (-1.9–3.7)	0.54		
> 5 years	179 (47.0)	39.2 (12.5)				
Missing	3 (0.8)					
**Duration of illness (months), n (%)**						
≤ 2 months	133 (34.9)	43.3 (14.6)	5.8 (2.9–8.7)	0.0001	Reference	
> 2 months	214 (56.2)	37.5 (12.7)			-5.09 (-8.03–-2.16)	0.001
Missing	34 (8.9)					
**PKDL grade, n (%)**						
Grade 2	49 (12.9)	39.3 (13.6)	-1.1 (-5.3–3.1)	0.61		
Grade 3	284 (74.5)	40.4 (13.8)				
Missing	48 (12.6)					
**Nutritional status, n (%)**						
Moderate/severe malnutrition	162 (42.5)	39.7 (14.3)	0.7 (-2.1–3.6)	0.62		
Mild malnutrition/ Normal status	183 (48.0)	38.9 (12.8)				
Missing	36 (9.5)					
**PKDL treatment regimen, n (%)**						
SSG	306 (80.3)	41.1 (13.3)	7.4 (3.9–10.9)	< 0.0001	Reference	0.005
SSG/PM	75 (19.7)	33.7 (15.5)			-5.13 (-8.73–-1.53)	

SD—Standard deviation; CI—Confidence interval; PKDL—Post-kala-azar dermal leishmaniasis; SSG—Sodium stibogluconate; PM–Paromomycin

Fifty patients (67%) among those treated with SSG/PM were cured within 30 days compared to 111 patients (36%) of those treated with SSG.

Sixteen (10.5%) of the 152 patients with a duration of illness at admission of ≤ 2 months and 53 (22.9%) of the 231 patients with a duration of illness at admission of > 2 months were treated with SSG/PM combination. The distribution of patients according to the duration of illness at admission was significantly different in the two treatment groups (p = 0.001). In cured patients with a documented treatment duration, the treatment duration was shorter among patients with a duration of illness at admission of > 2 months (-5.09 days; CI -8.03–-2.16; p = 0.001) as shown in Table 4.

Among cured patients with a documented treatment duration, 118 (88.7%) of the 133 patients with a duration of illness at admission of ≤ 2 months and 161 (75.2%) of the 214 patients with a duration of illness at admission of > 2 months were treated with SSG. In patients with a duration of illness of ≤ 2 months, the mean treatment duration was not significantly different in the two treatment groups; 43.6 (± 13.7) days in the SSG group compared to 40.7 (± 20.8) days in the SSG/PM group, (2.9 days; CI -4.9–10.9; p = 0.46). However, for patients with a duration of illness of > 2 months the mean treatment duration was significantly different in the two groups; 38.9 (± 12.0) days in the SSG group compared to 33.1 (± 13.8) days in the SSG/PM group, (5.8 days; CI 1.9–9.7; p = 0.0035).

## Discussion

We found that SSG/PM combination therapy provided significantly better outcomes than SSG monotherapy in PKDL patients in South Sudan. Patients were more likely to get cured, have a shorter treatment duration and they were less likely to default. Therefore this combination could be superior to SSG monotherapy and has a role in the treatment of severe PKDL.

The baseline clinical characteristics of the patients are similar to those reported in other studies in South Sudan [9]. Patients admitted with longer duration of illness healed faster than those with shorter duration of illness, indicating the natural healing process of PKDL. PKDL treatment accelerates this healing process [9].

In the study, the average weight of patients that were cured was 24 kg. The cost of SSG required for patients on SSG monotherapy treated for the mean treatment duration of 42 days is USD 54. The cost of SSG and PM required for patients on SSG/PM combination therapy treated for the mean treatment duration of 34 days is USD 52 [14]. The costs of drugs in the two treatment groups are relatively similar. However, the biggest component of treatment costs is hospitalisation. The shorter treatment with the combination therefore results in significantly lower hospitalisation costs. Hospitalisation (bed, food and shelter for the patient and a caretaker) is required for all patients, because they come from far and cannot be treated on ambulatory basis. Hospitalisation also enhances adherence to treatment.

SSG is administered by intramuscular injection, and can feasibly be provided at primary health care level, as it does not require cold chain. However, it is known to give rise to potentially serious toxicity (cardiotoxicity, nephrotoxicity, pancreatitis) and may even be fatal [17,18]. Toxicity is dose-dependent, and therefore the risk of toxic adverse events increases with the duration of treatment [17]. The risk of SSG-induced toxicity is therefore lower with the combination therapy as the PM in the treatment regimen significantly reduces the number of SSG doses required.

As PKDL is a non-fatal disease which usually does not lead to physical impairment, the treatment regimen should have a low risk/benefit ratio. In the absence of more suitable and safer drugs, SSG may be used for severe or complicated PKDL, however, it is not recommended for use in mild and moderate PKDL, where the benefits do not outweigh the potential harm to the patient. Therefore, public health VL control interventions aiming to reduce the human parasite reservoir by detecting and treating all PKDL patients should not be done with SSG-based regimens [9]. SSG should not be used in vulnerable patient groups with increased intolerability to SSG and at increased risk of severe (fatal) toxicity such as the HIV co-infected, pregnant and the very young and elderly patients [6,14,19,20]. Furthermore, SSG injections are painful and the longer the treatment duration, the more patients may decide to default from treatment. In the SSG group most patients absconded later during treatment. Defaulters weren’t interviewed to understand the reasons for defaulting as they were not retraced, however, a main reason for defaulting in this setting is said to be prolonged hospital stay whilst feeling well.

The PM dosage and duration was chosen as it had been shown to be safe and effective in combination with SSG in the treatment of VL [21]. PM toxicity (ototoxicity and nephrotoxicity) at a dose of 15 mg sulphate/kg/day is rare and is dose-related [22]. Therefore PM treatment should not exceed 17 days [14]. PM is also administered by painful intramuscular injections, and severe injection-site pain is a very common adverse event. However, PM does not require cold chain and can be implemented at primary health care level.

In general, the drugs currently available are not ideal for PKDL treatment because of high toxicity, high risk of resistance development, high prices, long treatment duration and often administered by injection [23,24]. In the absence of a safe, effective and cheap short course treatment for PKDL, treatment is only indicated for severe cases. For public health control interventions aimed at reducing the human parasite reservoir for transmission of *L*. *donovani*, drugs should meet a target product profile which makes it efficacious in a wide variety of the population (young, immunosuppressed patients etc.), safe (also in pregnancy, elderly and HIV), benefits should outweigh the risks, have an easy mode of administration (preferably oral) and of short course to promote patient compliance (ideally not lasting more than a week). Drugs should also be temperature stable and not require cold chain [23,24].

A main limitation of this study was that it was not a randomised trial, but an observational study carried out in different health care settings. The clinical diagnosis and follow up of patients treated with the SSG/PM combination was done by medical doctors in secondary health care facilities, and patients treated with SSG monotherapy were diagnosed and followed up by nurses or CHWs in primary health care facilities. There are many possible differentials of PKDL in South Sudan among which the most common are leprosy, *miliaria rubra*, measles, acne, scabies, tribal markings and lupus vulgaris [3]. Medical doctors are more skilled than CHWs so this may have introduced observer bias. However, because patients were only admitted with severe PKDL, characterised by a typical maculo-popular rash often involving the mucosa and a history of VL within the last six months, the observer bias is assumed to be minimal. Response to treatment is a marker of a correct PKDL diagnosis. Other limitations are that the VL treatment regimen and the time lag between the end of VL treatment and the appearance of PKDL lesions were not systematically collected. Furthermore, working in remote, extremely resource-limited and war-affected settings without possibility of installing medical equipment and availability of qualified and experienced staff, we were not able to carry out routine cardiac, pancreatic, renal and hepatic function tests to document potential adverse events. During the study period, systematic HIV screening was not yet done, however, the HIV prevalence in South Sudanese population is estimated at less than 1% [25], especially in young children, who make up the majority of this PKDL patient cohort.

Further research to improve efficacy and safety of treatment using new combinations of currently available drugs are a priority. Immuno-chemotherapy using alum-precipitated autoclaved *L*. *major* plus Bacille Calmette-Guérin (BCG) used in combination with SSG was shown to be effective in the treatment of persistent PKDL [26]. More studies on different immune-chemotherapeutic modalities are needed.

PKDL cure is currently based on clinical resolution of skin lesions. However, parasitological cure or effective immune-modulation may precede clinical healing of lesions. Therefore identification of clinical and/or laboratory markers for PKDL to predict the prognosis of healing (rapid self-cure or need for treatment) are required. Additionally, clinical and/or laboratory markers that predict parasitological cure after treatment are also required.

There are many knowledge gaps around the epidemiology, pathogenesis, susceptibility, diagnosis, and treatment of PKDL. However, funding for PKDL research has been severely neglected [24]. This is mainly because, unlike VL, PKDL is not a fatal or debilitating condition and in most cases self-healing. Nevertheless, because PKDL may play an important role in the transmission of *Leishmania donovani* parasites, research to answer the knowledge gaps can play a key role in the development of strategies to control VL, which is a deadly disease.
